# Understanding East–West Cultural Differences on Perceived Compensation Fairness Among Executives: From a Neuroscience Perspective

**DOI:** 10.3389/fpsyg.2021.815641

**Published:** 2022-02-02

**Authors:** Fan Yu, Ying Zhao, Jianfeng Yao, Massimiliano Farina Briamonte, Sofia Profita, Yuhan Liu

**Affiliations:** ^1^Business School, Yunnan University of Finance and Economics, Kunming, China; ^2^Zhonghua Vocational College, Yunnan University of Finance and Economics, Kunming, China; ^3^Department of Economics and Business, University of Sassari, Sassari, Italy; ^4^Financial Sponsors Group of Santander Bank, New York, NY, United States; ^5^Business College, Beijing Union University, Beijing, China

**Keywords:** cognitive differences, perceived compensation fairness, Confucian culture, Western culture, cultural differences, cognitive neuroscience, innovation motivation

## Abstract

Cognitive neuroscience research has found that individuals from different cultures have different neural responses and emotional perceptions. Differences in executives’ perception of external pay gaps in different cultures can affect their work attitudes and behavior. In this study, we explore the direct relationship between executive compensation fairness and executive innovation motivation. We also investigate the moderating effects of Confucian culture and western culture between executive compensation fairness and executive innovation motivation. Data were collected from the Chinese listed firms from 2011 to 2019 and test the relationship using the fixed-effect panel regression models. The results demonstrate that executive compensation fairness positively influences the executive innovation motivation. This effect is more pronounced in Confucian culture regions, while Western culture weakens this effect. The findings of this study confirmed that executive compensation fairness, provide a cross-cultural comparison for compensation research, validate the findings of cultural neuroscience, and provide useful insights into the research of common prosperity. To improve the corporate compensation structure, it is necessary to consider the relative pay equity with firms in the same region and the influence of corporate culture.

## Introduction

China’s common prosperity, which proposes a vision toward a more equitable economic and social system, is currently a hot topic across the world. The increase in income inequality among corporate employees in recent years is mainly brought about by the pay disparity between different firms (Song et al., 2019). Therefore, in order to promote common prosperity, the trend is to reduce the pay disparity between firms and to pursue compensation fairness. For firms, it becomes a question of whether this will facilitate or hinder their development.

According to tournament theory, the pay disparity is seen as a tournament incentive (Lazear and Rosen, 1981). The external pay disparity for executives is then described as an incentive for industry tournaments or local tournaments. Many findings from empirical studies suggest that the pay gap has a motivating effect on executives (Coles et al., 2018; Huang et al., 2019; Ma et al., 2019). Some famous scholars demonstrate that prospective equity studies have argued that equity theory focuses on people’s psychological perceptions of income fairness. Therefore, a significant pay gap may trigger psychological perceptions of unfairness (Adams, 1963). Similarly, the scholars of social preference also emphasize people’s pursuit of fairness. These theories are probably sound, and maybe they ignored the heterogeneity of corporate cultural contexts.

Previous studies on cultural neuroscience suggest that different cultural backgrounds bring systematically different experiences to individuals. The neural response areas corresponding to the typical behavior with the cultural brand will be activated repeatedly (Kitayama and Uskul, 2011; Han et al., 2013). Prior studies showed that cultural background influences differences in human neural mechanisms such as attention (Lewis et al., 2008), self-representation (Chiao et al., 2010), and self-enhancement (Hampton and Varnum, 2018). People have different neural responses under various cultural contexts (Varnum and Hampton, 2017). From this perspective, we may offer possible explanations for the academic debate on the impact of the pay disparity on executive behavior. In a culture concerned with collectivism and fairness, corporate executive behavior is better explained by equity theory; while in a culture concerned with individualism and efficiency, corporate executive behavior is better explained by tournament theory. We amplify this cultural difference between firms into an East–West cultural difference for our research purposes.

Cultural differences between the United States and East Asia shape the different neural response ways of regulating emotions (Hampton et al., 2021). This leads to variability in the emotional experiences and responses of the two populations (Chin et al., 2021a). Confucianism is one of the most influential philosophies from ancient China, which laid the foundation for much of Chinese culture. It is concerned with inner virtue, morality, and values. In China, the idea of systemic equity is dominant due to Confucianism’s widespread and far-reaching influence (Ren, 2012). The importance of equity is emphasized in Confucius, Mencius, and Xunzi. From ancient times to the present, the Confucian concept of fairness has been deeply rooted in people’s hearts and minds, thus promoting fairness awareness. In other words, in a Confucian cultural context, people’s neural response areas to perceptions of fairness are activated more often. People thus generate relatively stronger neural response mechanisms. Referring to neural responses, innovation motivation is an essential representation of neural responses to corporate executives’ perceptions of compensation fairness (Mas, 2006; Torre et al., 2015). In the context of Western individualistic cultures, many empirical studies have demonstrated that the external pay disparity of corporate executives is positively related to their innovation motivation (Coles et al., 2018; Ma et al., 2019). It is necessary to verify the existence of opposed relationships in Eastern cultures.

Therefore, we gather data from the Chinese listed firms from 2008 to 2019 in this study. We merged financial data from China Stock Market and Accounting Research Database (CSMAR) and Confucian culture and R&D data from the Chinese Research Data Service Platform (CNRDS). We obtained 12-year unbalanced panel data with 14,296 observations. From the perspective of cognitive neuroscience, we explore what perceptions and responses will be generated by corporate executives’ perceived external pay gap on the innovation motivation using fixed-effect panel data regression models. The research conclusion has the following two aspects: First, it clarifies the mechanisms of the influence of compensation on executives’ innovation motivation and corporate innovation commitment. Thus, it enriches the study of incentive contracts and corporate innovation. Second, it explains the theoretical applicability of compensation fairness in different cultural contexts and provides a cross-cultural comparison for compensation fairness research. At the same time, the research findings also provide theoretical references and suggestions for firms to develop reasonable compensation systems.

The structure of this study is as follows: Second part explains the “Theory and Hypotheses.” The third part entails the details about the “Research Methods”. In the fourth part, the “Results” are described in detail. The fourth part explains the “Discussion,” including the “Theoretical and Practical Implications”. The last part is defining the “Limitations and Future Research Directions”.

## Theory and Hypotheses

### Executive Compensation Fairness and Executive Innovation Motivation

Psychologists have used event-related potential, magnetoencephalography, and functional magnetic resonance imaging (fMRI) to deeply examine the brain mechanisms of fair decision-making (Stallen and Sanfey, 2013; Declerck et al., 2014). The fMRI results find that regions related to reward information processing such as the ventral striatum, ventral medial prefrontal, and orbitofrontal cortex are activated (Chen et al., 2017). This result suggests that a fair distribution proposal can act as a reward to bring pleasure emotions to people. This finding supports the equity theory model of social preference theory: Individuals consider their total income and relative income to others in social decision-making. And the state of equity itself is a reward (Cheng et al., 2017). In contrast, when people are faced with an unfair distribution plan, activation of the forebrain insula shows disgust or anger (Tricomi et al., 2010). The results of cognitive neuroscience experiments support the equity theory.

People self-evaluate by comparing themselves with others without direct natural standards (Festinger, 1954). This leads to the perception of fairness (Ambrose et al., 1991). Employees’ job satisfaction in a firm is highly correlated with the equity of the treatment they receive. Compensation is financial gain that a worker in business makes to the organization for their mental or physical work. It is a monetary income in exchange for using one’s skills, experience, and abilities (Khan et al., 2021). In turn, compensation fairness is the most critical component of treatment equity (Dittrich et al., 1985). People will generate a psychological perception of expectations, triggering a series of negative behaviors (Adams, 1963). However, the social rewards from compensation fairness can sometimes outweigh the pleasure from purely material rewards (Ruff and Fehr, 2014). The compensation fairness of corporate executives can be divided into internal equity and external equity. External equity, the difference in compensation between executives and those in the same region, industry, or other related firms, has a more significant impact on executive satisfaction than internal compensation fairness (Scholl et al., 1987).

It is crucial for firms to pursue technological innovation to maintain their long-term competitive advantage continuously (Wang and Deng, 2021). Executives play a decisive role in strategic planning and directly influence corporate innovation (Holmstrom, 1989; Rong and Wang, 2021). Environmental factors impact work motivation (Yang and Wu, 2021), so a fair working environment raises the level of effort. A key factor affecting executives’ motivation is their perception of compensation fairness (Torre et al., 2015). Executives’ innovative motivation determines the firm’s creative activity intensity (Duan et al., 2021). In practical terms, executives’ perceptions of compensation fairness can motivate them to use their management talents (Scarpa et al., 2021). Then they will generate a stronger innovation motivation in the firm’s long-term interest. In comparison, perceptions of compensation unfairness will trigger negative emotions in executives and reduce incentives to innovate. In a Western cultural context, the tournament incentive effect of the pay gap may diminish these effects and motivate executives to innovate (Coles et al., 2018; Ma et al., 2019). Psychological studies have shown that Chinese people pay more attention and importance to distributive justice in Confucian cultural contexts than people in Western countries (Kim and Leung, 2007). In the context of a Confucian culture that values fairness, the motivation of Chinese executives to innovate can be swayed by compensation fairness. Therefore, we propose the following hypothesis:

*Hypothesis 1: Executive compensation fairness positively impacts on executive innovation motivation in China.*

In terms of direction, executives’ perceptions of compensation fairness can be further divided into upward comparisons (comparisons with those who are paid more) and downward comparisons (comparisons with those who are paid less). Among the three models of social preferences, the outcome-based difference aversion model assumes that people are consistently less satisfied with an unfair situation. Moreover, the loss of utility in disadvantaged unfairness is greater than in advantaged unfairness (Fehr and Schmidt, 1999; Tricomi et al., 2010). Since the achievement orientation of executives drives a preference for upward comparisons (Park and Westphal, 2013). Therefore, when an executive’s compensation is lower than the external reference point, perceptions of unfairness and jealousy will reduce executive motivation and increase executive negligence and other irresponsible behaviors (Mas, 2006). When executives are paid less than others, the negative pay gap triggers stronger negative emotional and behavioral responses and significantly impacts innovation motivation. Therefore, based on hypothesis 1, we propose the following hypothesis:

*Hypothesis 2: Executive’s perceptions of compensation fairness strongly impact on executive innovation motivation when at a compensation disadvantage.*

### Moderating Effects of Cultural Contexts

People have different neural responses under various cultural contexts (Varnum and Hampton, 2017). Cultural differences lead people to make other decisions in their economic activities (Guiso et al., 2006). It is undeniable that Confucianism has profoundly influenced Chinese cognition and behavior (Tu, 2005; Chin et al., 2021b). As Confucius demonstrate that the chief of a state or a family need not care for scarcity but inequality, nor for poverty but security (Xu, 2012). Therefore, he believed that the distribution of social wealth should be reasonable and regulated in a balanced way so that people would have no grievances. Mencius and Xunzi then inherited and carried forward the Confucian idea of equalization. Dong Zhongshu, a famous Confucianist and politician in the Han Dynasty, elevated the idea of equity to the strategic level of harmonious social development and long-term national security, making the idea of equity a fundamental value of Confucianism. It can be seen that the Confucian concept of fairness has played an essential role in history and contributed to people’s fairness awareness. Since executive compensation fairness positively impacts innovation motivation in the context of Confucian culture, Both the incentive effect of compensation fairness on executives’ innovation motivation and the hindering effect of compensation unfairness are enhanced by Confucian cultural influence. So we propose the following hypothesis:

*Hypothesis 3: Confucian culture context moderate between executive compensation fairness on executive innovation motivation.*

However, the Western culture regards wealth and success as the result of individual effort. This determines their perception of compensation fairness (Alesina and Angeletos, 2005). In this context, the pay gap reflects unique talent and effort differences. It is less likely to raise perceptions of equity but more often seen as an incentive which is described as tournament incentives. From this understanding, Western scholars believe that tournament incentives can motivate people to work harder to pursue a better position (Kale et al., 2009) and both industry tournament incentives (Coles et al., 2018) and local tournament incentives (Ma et al., 2019) have similar facilitating effects. Yet, other studies suggest that executives may take improper means (Bebchuk et al., 2011; Haß et al., 2015). In general, the pay gap is perceived as a “goal to strive for” in the Western culture, and the psychological perception of unfairness is weaker. Therefore, compensation fairness has less impact on executives’ innovation motivation. To summarize, we propose the following hypothesis:

*Hypothesis 4: Western cultural context moderate between executive compensation fairness on executive innovation motivation.*

**FIGURE 1:**
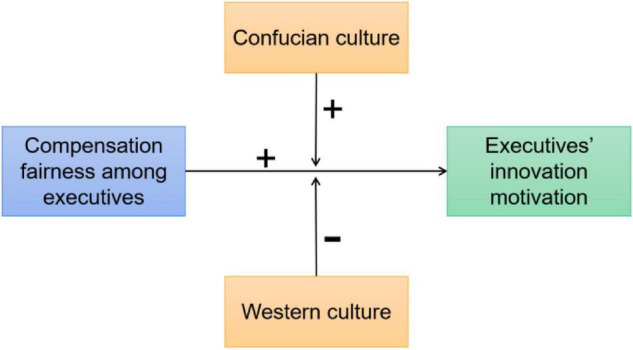
Conceptual model.

Based on the above literature and discussion, we proposed the below-mentioned research model of this study. Therefore, Figure 1 presents the conceptual model of this study.

## Research Methods

### Research Approach

A quantitative approach was taken in the research design in order to test the proposed research hypotheses. The quantitative data were collected from the Chinese listed firms from 2011 to 2019. We used this research approach because it is a popular method and relatively low cost as compared to other research methods.

### Sampling and Data Collection

The target population of this study was the corporate executives such as directors, supervisors, and CEOs of Chinese listed firms. Corporate executives can more easily access the compensation of management of other listed firms and better understand their compensation fairness. Limited by data availability, China’s A-share listed firms from 2008 to 2019 are selected as the sample in this study. The firms’ basic information and financial data are sourced from China Stock Market and Accounting Research Database (CSMAR). The Confucian culture and R&D data are from the Chinese Research Data Service Platform (CNRDS). We merged these two data sources according to each record’s stock code and the accounting year. These records are phased out according to the following criteria: (1) Firms belonging to financial industries; (2) Special treatment stocks with abnormal operating conditions (ST Stocks); (3) Firms that have never applied for patents during this period; (4) Firms with missing severe data on critical variables. After processing, we obtained 12-year unbalanced panel data for 2,928 listed firms with 14,296 observations. To avoid the effect of extreme values, we winsorized all continuous-type variables at 1 and 99% quartiles.

### Measures

Combined with the previous analysis, the basic regression model is constructed in this paper as follows:


(1) $$RDSpd_{i,t}=\alpha_{0}+\alpha_{1}Cf_{i,t}+\alpha_{\text{j}}\sum Controls_{i,t}^{j}+\varepsilon$$

The dependent variable is executive innovation motivation. Patents and R&D are simultaneously used to measure innovation in the literature (Yu et al., 2021). According to the Upper Echelon Theory, corporate executives acquire and allocate resources according to the internal and external environment (Romer, 1986; Rasool et al., 2019). So the executives’ innovation motivation can be measured by the level of funds invested in innovation activities. The proxy is the ratio of R&D input to operating revenues (RDSpd), which is presented as a percentage.

The independent variable is compensation fairness (Cf). The primary measurement methods of compensation fairness are pay gap calculation, questionnaire survey, regression model residual, and relative quantile calculation methods. The questionnaire survey method is highly subjective (Tekleab et al., 2005). The pay gap method considers only numerical differences without focusing on other factors that trigger the differences. The regression model calculates the residuals by constructing a model of executive compensation decisions, but its validity is strongly influenced by choice of variables (Core et al., 1999). We choose the relative quantile method to express compensation fairness (Song et al., 2019). The difference between executive compensation and the highest local executive compensation to the firm performance (ROA) is calculated. The higher the value, the lower the compensation fairness.

∑Controls is the control variables. Firm-level financial characteristics can influence innovation input (Yu et al., 2021). So we control for total assets (Size), assets liabilities ratio (Lev), profitability (ROE), and growth potential (Growth) in the regression model. Corporate governance is the institutional basis for corporate innovation (Belloc, 2012). So we include the concentration of equity (Shr1), whether two positions are combined (Dual), the percentage of independent directors (InDir), and the number of board of directors (Dir) as control variables (Allen et al., 2015).

## Results and Analysis

### Descriptive Statistics

The results of descriptive statistics for the main variables in this manuscript are shown in Table 1. The average proportion of innovation investment in the operating income of the sample firms is 4.47%. Executive compensation fairness values range from −7.43 to 2.46, reflecting a pay gap between local firms with a minimum ratio of 0.001 and a maximum ratio of 11.69 to the ROA gap. There is an unfair phenomenon in the executive compensation in the same region, and the maximum pay gap in executives is 38 times. The average shareholding of the first largest shareholder is about one-third, indicating a high concentration of equity. The board of directors consists of eight members on average. The average proportion of independent directors to the board of directors is about one-third, just the minimum standard set by the China Securities Regulatory Commission (CSRC).

**TABLE 1:** Descriptive statistical results.

Variables	*N*	Mean	SD	Median	Min	Max
RDSpd	14,296	4.47	4.24	3.56	0.02	25.37
Cf	14,296	0.06	1.17	0.08	–7.43	2.46
Size	14,296	22.02	1.21	21.84	19.92	25.72
Lev	14,296	0.39	0.19	0.37	0.05	0.87
ROE	14,296	0.09	0.07	0.08	–0.64	0.33
Growth	14,296	0.19	0.37	0.12	–0.48	2.29
Shr1	14,296	34.56	14.14	32.97	9.00	71.77
Dual	14,296	0.29	0.46	0.00	0.00	1.00
InDir	14,296	0.37	0.05	0.33	0.33	0.57
Dir	14,296	2.12	0.19	2.20	1.61	2.64

### Regression Analysis

#### Regression Result

Following the Hausman test, a fixed-effects model was applied to the sample data regression, and the regression results are shown in Table 2. The Cf coefficient in column (1) is significantly negative, indicating that the lower the compensation fairness is, the less the corporate invests in innovation. That is to say, executives’ perception of compensation fairness is positively related to innovation motivation. This verifies hypothesis H1. In column (2), we select observations with Cf values greater than or equal to zero, i.e., the compensation disadvantaged sample group where the local pay gap is greater than the performance gap. The Cf value is significantly negative and becomes more prominent in absolute value. That is to say when executives perceive that their compensation is lower than the “deserved level,” the perception of unfairness will significantly reduce the innovation motivation, which supports H2. Correspondingly, column (3) is the strong side of the compensation sample. Although the Cf coefficient is insignificant, the executives do not increase their innovation motivation because their compensation is higher than the “deserved level.” This is a sense, confirms their preference for equity.

**TABLE 2:** Regression results.

Variables	(1) Entire sample	(2) Negative sample	(3) Positive sample
	
	RDSpd	RDSpd	RDSpd
Cf	−0.090**	−0.274***	−0.042
	(−2.37)	(−3.51)	(−0.80)
Size	0.070	0.049	0.090
	(0.61)	(0.39)	(0.47)
Lev	−2.677***	−2.581***	−1.772***
	(−7.43)	(−5.85)	(−2.99)
ROE	−2.038***	−2.959***	−0.400
	(−2.85)	(−2.77)	(−0.49)
Growth	−0.555***	−0.584***	−0.492***
	(−9.63)	(−8.71)	(−4.21)
Shr1	−0.004	−0.008	−0.005
	(−0.71)	(−1.33)	(−0.51)
Dual	0.072	0.077	0.090
	(0.86)	(0.79)	(0.68)
InDir	−1.224	−1.232	−1.176
	(−1.33)	(−1.03)	(−0.96)
Dir	0.417	0.479	0.431
	(1.21)	(1.10)	(1.00)
_cons	2.696	3.717	1.620
	(1.10)	(1.27)	(0.39)
Control year/industry	Yes	Yes	Yes
*N*	14,296	8,258	6,038
Adj *R*^2^	0.072	0.083	0.075

t statistics in parentheses, *p < 0.1; **p < 0.05; ***p < 0.01.

As for the control variables, firms with better performance in assets and liabilities, growth potential, and return on net assets have less R&D investment. In other words, such firms are relatively less motivated to innovate. Corporate governance-related control variables are generally not related to corporate innovation, probably because the overall level of corporate governance still needs to be improved.

#### The Influence of Confucian Culture

Based on existing studies, the influence of Confucian culture in a region is measured by the number of Confucian temples remaining in the region where the firms is located. Accordingly, all country regions are divided into three groups according to Confucian cultural influence. The group regression results are shown in Table 3. Columns (1–3) examine the entire sample of firms, and columns (4–6) test the sample group of compensation disadvantaged parties. The coefficient of Cf in the last three columns is significantly negative and significantly more prominent in absolute value than the first three columns. This indicates that the effect of compensation fairness on innovation motivation and the perceived impact of unfairness is more significant on the compensation disadvantaged side. Hypotheses H1 and H2 conclusions are robust. Comparing the firm samples from high, medium, and low Confucian influence regions, it is clear that the influence of compensation fairness on innovation investment is more substantial for corporate executives in a culture with a more significant Confucian impact. That is to say, Confucian culture makes executives have a stronger preference for equity and will react more strongly to unfairness. Thus, Hypothesis H3 is supported.

**TABLE 3:** Regression results based on the regression of Confucian cultural influence group.

Variables	(1)	(2)	(3)	(4)	(5)	(6)

	Entire sample			Negative sample		
	Low Confucian influence regions	Medium Confucian influence regions	High Confucian influence regions	Low Confucian influence regions	Medium Confucian influence regions	High Confucian influence regions

	RDSpd	RDSpd	RDSpd	RDSpd	RDSpd	RDSpd
Cf	−0.082	−0.081	−0.127*	−0.203*	−0.352***	−0.390***
	(−1.64)	(−1.63)	(−1.83)	(−1.77)	(−2.83)	(−3.42)
Size	0.317*	0.188	−0.200	0.126	−0.187	0.077
	(1.96)	(0.67)	(−1.04)	(0.71)	(−0.64)	(0.33)
Lev	−2.651***	−2.041***	−3.146***	−2.878***	−1.497**	−3.090***
	(−4.26)	(−2.67)	(−5.41)	(−3.57)	(−1.97)	(−4.86)
ROE	−1.728***	−1.464	−1.114	−2.422***	−1.741*	−1.821
	(−2.60)	(−1.42)	(−1.17)	(−2.87)	(−1.66)	(−1.62)
Growth	−0.570***	−0.642***	−0.544***	−0.712***	−0.540***	−0.557***
	(−7.05)	(−5.32)	(−5.11)	(−6.71)	(−4.24)	(−4.23)
Shr1	−0.018**	0.012	0.009	−0.015*	−0.013	0.002
	(−2.02)	(0.86)	(0.93)	(−1.68)	(−0.76)	(0.15)
Dual	0.023	−0.160	0.235*	−0.003	0.084	0.216
	(0.20)	(−0.75)	(1.93)	(−0.02)	(0.39)	(1.37)
InDir	0.096	−3.921**	−1.439	−0.333	−5.308***	0.129
	(0.06)	(−2.09)	(−1.12)	(−0.15)	(−2.65)	(0.08)
Dir	0.784	−0.925	0.559	0.425	−0.995	1.270**
	(1.19)	(−1.40)	(1.47)	(0.48)	(−1.53)	(2.46)
_cons	−3.181	3.862	7.610*	2.940	12.157*	0.567
	(−0.89)	(0.72)	(1.83)	(0.74)	(1.76)	(0.11)
Control variables	Yes	Yes	Yes	Yes	Yes	Yes
Control year/industry	Yes	Yes	Yes	Yes	Yes	Yes
*N*	5983	2863	5093	3648	1606	2775
Adj *R*^2^	0.072	0.074	0.081	0.082	0.113	0.084

t statistics in parentheses, *p < 0.1, **p < 0.05, ***p < 0.01.

#### The Influence of Western Culture

The degree of Western cultural influence is judged by the overseas experience of the Chairman or CEO. If the Chairman or CEO has an overseas study or work experience, the firm is considered to have a strong influence on Western culture. See Table 4 for the group regression results according to high and low Western cultural impact. Columns (1) and (2) examine the entire sample of firms, and columns (3) and (4) test the compensation disadvantaged sample groups. Hypotheses H1 and H2 are robustness. As seen in columns (2) and (4), the effect of executive compensation fairness perception on innovation motivation is more negligible in the sample group where the Chairman or CEO has overseas experience. That is to say, the preference for equity among executives in Confucian culture is reduced when firms are influenced by Western culture. Thus, Hypothesis H4 is approved.

**TABLE 4:** Regression results based on the regression of Chairman and CEO overseas experience group.

Variables	(1)	(2)	(3)	(4)

	Entire sample		Negative sample	
	Chairman or CEO with overseas experience	Chairman or CEO without overseas experience	Chairman or CEO with overseas experience	Chairman or CEO without overseas experience

	RDSpd	RDSpd	RDSpd	RDSpd
Cf	−0.008	−0.093**	−0.127*	−0.203*
	(−0.05)	(−2.44)	(−1.83)	(−1.77)
Size	−0.428	0.063	−0.200	0.126
	(−0.70)	(0.56)	(−1.04)	(0.71)
Lev	−2.460	−2.637***	−3.146***	−2.878***
	(−1.30)	(−7.46)	(−5.41)	(−3.57)
ROE	−2.175	−2.137***	−1.114	−2.422***
	(−1.35)	(−2.77)	(−1.17)	(−2.87)
Growth	−0.449**	−0.551***	−0.544***	−0.712***
	(−2.58)	(−8.92)	(−5.11)	(−6.71)
Shr1	−0.001	−0.007	0.009	−0.015*
	(−0.06)	(−1.22)	(0.93)	(−1.68)
Dual	0.132	0.077	0.235*	−0.003
	(0.52)	(0.88)	(1.93)	(−0.02)
InDir	2.585	−1.360	−1.439	−0.333
	(0.67)	(−1.44)	(−1.12)	(−0.15)
Dir	2.236**	0.201	0.559	0.425
	(2.11)	(0.57)	(1.47)	(0.48)
_cons	7.237	3.447	7.610*	2.940
	(0.56)	(1.42)	(1.83)	(0.74)
Control variables	Yes	Yes	Yes	Yes
Control year/industry	Yes	Yes	Yes	Yes
*N*	1,167	13,059	5,093	3,648
Adj *R*^2^	0.074	0.073	0.081	0.082

t statistics in parentheses, *p < 0.1, **p < 0.05, ***p < 0.01.

### Robustness Tests

Under a narrower definition of executive, we focus on the impact of CEO compensation fairness and innovative behavior. The indicator of the external pay gap for executives is chosen as the CEO regional pay gap and is calculated as the ratio of the highest CEO compensation in the region to the CEO compensation in the firm. Compensation unfairness is expressed as the ratio of the CEO regional pay gap to the ROA gap. In addition, replace the indicator of Confucian cultural influence, and regroup the regression as measured by the total number of Ming and Qing scholars in the region. Replace the indicator of Western cultural impact with the index of regional openness to judge and regroup the regression. All the above results indicate that the findings of this paper are robust.

## Discussion

The idea of fairness is one of the foundations of the Confucian value system. It carries the idea that “Goals of self and others can be unified; thus the world can be harmonized.” Confucianism is rooted deep in the Chinese spirit (Tu, 2005). Thus, Chinese people pay more attention to compensation fairness than Western countries (Kim and Leung, 2007). In Western culture, many empirical studies have demonstrated that the external pay disparity of corporate executives is positively related to their innovation motivation (Coles et al., 2018; Ma et al., 2019; Rasool et al., 2021). Our study tries to verify the existence of opposed relationships in the context of Eastern cultures and finds that: The executive pay gap, which is generally regarded as a tournament incentive in Western culture, can be used to estimate executives’ psychological perceptions of compensation fairness in China. Executive compensation fairness is generally positively associated with innovation motivation in China, especially when at a compensation disadvantage. There are different contextual effects of cultural differences on the above relationships. First, corporate executives who are more influenced by Eastern Confucian culture have a more vital role of compensation fairness perception on innovation motivation. Second, corporate executives who are more influenced by Western culture have a weaker role of perceived compensation fairness on innovation motivation.

### Theoretical Contributions

This study has three main theoretical contributions. First, cognitive neuroscience experiments have found that cultural differences can shape different neural responses, which leads to differences in people’s emotional perception and behavioral responses from other cultures when they face similar events. Second, in this study, we suggest that the cultural contexts influence the two theoretical explanations of the pay gap. Tournament theory applies in the Western cultural context, and equity theory lay in the Confucian cultural context. Third, this study provides useful insights into the research of the idea of common prosperity at the corporate level. To address the fundamental relationships between “efficiency and fairness”, reducing unjustified pay disparity can achieve both goals at the same time.

### Practical Implications

The findings of this study indicate some practical implications that could increase executive innovation motivation. First, organizations should organize some healthy activities for their employees (e.g., family fairs and sports events) as well as revise the pay structure of the executive positions. Second, directors, supervisors, and CEOs of Chinese organizations need to identify key employees who have innovative behavior and then provide them with soft skills, e.g., relationship management, work time and stress management, and personality development. These steps will enhance the innovations in Chinese organizations. Third, Chinese organizations need to design executive compensation fairness that will enhance the motivation among Chinese organizations. Finally, top-level management should encourage a positive work environment and provide more incentives and special allowances. These steps can help to increase executive innovation motivation.

### Limitations and Future Directions

This study has some limitations and future research directions. The first limitation is that the respondents were selected only from the Chinese listed firms. This is a limitation in terms of generalizability under the influence of cultural and contextual biases. Therefore, to generalize the results in the future, such kind of study can be investigated in the developed or underdeveloped countries. The second limitation was the sample size of the study, which may influence the generalizability of the results. However, to overcome these limitations, the research has undertaken used certain precautions. To eliminate the cultural and contextual biases, the results of the research have been interpreted in li1ne with the relevant studies. Third, the future researcher can investigate the mediating effect of job satisfaction to test the relationship between executive compensation fairness and executive innovation motivation. This will help the organizations reduce the turnover of the organization. This direction will improve the motivation level of the employees that will also positively affect the organizational innovations.

## Data Availability Statement

The original contributions presented in the study are included in the article/supplementary material, further inquiries can be directed to the corresponding author.

## Author Contributions

FY designed the research and wrote the main part of the manuscript. YZ and SP collected the data and wrote the “Research Methods” section. JY and MF offered modification suggestions and helped translating the manuscript. YL provided guidance throughout the entire research process. All authors contributed to the article and approved the submitted version.

## Conflict of Interest

SP was employed by company Financial Sponsors Group of Santander Bank. The remaining authors declare that the research was conducted in the absence of any commercial or financial relationships that could be construed as a potential conflict of interest.

## Publisher’s Note

All claims expressed in this article are solely those of the authors and do not necessarily represent those of their affiliated organizations, or those of the publisher, the editors and the reviewers. Any product that may be evaluated in this article, or claim that may be made by its manufacturer, is not guaranteed or endorsed by the publisher.

## References

[B1] Adams J. S. (1963). Towards an understanding of inequity. *J. Abnorm. Soc. Psychol.* 67 422–436. 10.1037/h0040968 14081885

[B2] Alesina A. Angeletos G.-M. (2005). Fairness and Redistribution. *Am. Econ. Rev.* 95 960–980. 10.1257/0002828054825655

[B3] Allen M. R. Adomdza G. K. Meyer M. H. (2015). Managing for innovation: managerial control and employee level outcomes. *J. Bus. Res.* 68 371–379. 10.1016/j.jbusres.2014.06.021

[B4] Ambrose M. L. Harland L. K. Kulik C. T. (1991). Influence of social comparisons on perceptions of organizational fairness. *J. Appl. Psychol.* 76 239–246. 10.1037/0021-9010.76.2.239

[B5] Bebchuk L. A. Cremers K. J. M. Peyer U. C. (2011). The CEO pay slice. *J. Financ. Econ.* 102 199–221. 10.1016/j.jfineco.2011.05.006

[B6] Belloc F. (2012). Corporate Governance and Innovation: a Survey. *J. Econ. Surv.* 26 835–864. 10.1111/j.1467-6419.2011.00681.x

[B7] Chen Y.-H. Chen Y.-C. Kuo W.-J. Kan K. Yang C. C. Yen N.-S. (2017). Strategic Motives Drive Proposers to Offer Fairly in Ultimatum Games: an fMRI Study. *Sci. Rep.* 7:527. 10.1038/s41598-017-00608-8 28373714 PMC5428836

[B8] Cheng X. Zheng L. Li L. Zheng Y. Guo X. Yang G. (2017). Anterior insula signals inequalities in a modified Ultimatum Game. *Neuroscience* 348 126–134. 10.1016/j.neuroscience.2017.02.023 28223239

[B9] Chiao J. Y. Harada T. Komeda H. Li Z. Mano Y. Saito D. (2010). Dynamic Cultural Influences on Neural Representations of the Self. *J. Cogn. Neurosci.* 22 1–11. 10.1162/jocn.2009.21192 19199421

[B10] Chin T. Meng J. Wang S. Shi Y. Zhang J. (2021a). Cross-cultural metacognition as a prior for humanitarian knowledge: when cultures collide in global health emergencies. *J. Knowl. Manag.* 10.1108/JKM-10-2020-0787 [Epub ahead of print].

[B11] Chin T. Shi Y. Rowley C. Meng J. (2021b). Confucian business model canvas in the Asia Pacific: a Yin-Yang harmony cognition to value creation and innovation. *Asia Pac. Bus. Rev.* 27 342–358. 10.1080/13602381.2020.1795481

[B12] Coles J. L. Li Z. Wang A. Y. (2018). Industry Tournament Incentives. *Rev. Financ. Stud.* 31 1418–1459. 10.1093/rfs/hhx064

[B13] Core J. E. Holthausen R. W. Larcker D. F. (1999). Corporate governance, chief executive officer compensation, and firm performance. *J. Financ. Econ.* 51 371–406. 10.1016/S0304-405X(98)00058-0

[B14] Declerck C. H. Boone C. Kiyonari T. (2014). The effect of oxytocin on cooperation in a prisoner’s dilemma depends on the social context and a person’s social value orientation. *Soc. Cogn. Affect. Neurosci.* 9 802–809. 10.1093/scan/nst040 23588271 PMC4040087

[B15] Dittrich J. E. Daniel Couger J. Zawacki R. A. (1985). Perceptions of equity, job satisfaction, and intention to quit among data processing personnel. *Inf. Manag.* 9 67–75. 10.1016/0378-7206(85)90028-X

[B16] Duan Y. Huang L. Luo X. Cheng T. C. E. Liu H. (2021). The moderating effect of absorptive capacity on the technology search and innovation quality relationship in high-tech manufacturing firms. *J. Eng. Technol. Manage.* 62:101656. 10.1016/j.jengtecman.2021.101656

[B17] Fehr E. Schmidt K. M. (1999). A Theory of Fairness, Competition, and Cooperation. *Q. J. Econ.* 114 817–868. 10.1162/003355399556151

[B18] Festinger L. (1954). A theory of social comparison processes. *Hum. Relat.* 7 117–140. 10.1177/001872675400700202

[B19] Guiso L. Sapienza P. Zingales L. (2006). Does Culture Affect Economic Outcomes? *J. Econ. Perspect.* 20 23–48. 10.1257/jep.20.2.23

[B20] Hampton R. S. Kwon J. Y. Varnum M. E. W. (2021). Variations in the regulation of affective neural responses across three cultures. *Emotion* 21 283–296. 10.1037/emo0000711 31815497

[B21] Hampton R. S. Varnum M. E. W. (2018). Do cultures vary in self-enhancement? ERP, behavioral, and self-report evidence. *Soc. Neurosci.* 13 566–578. 10.1080/17470919.2017.1361471 28749312

[B22] Han S. Northoff G. Vogeley K. Wexler B. E. Kitayama S. Varnum M. E. W. (2013). A cultural neuroscience approach to the biosocial nature of the human brain. *Annu. Rev. Psychol.* 64 335–359. 10.1146/annurev-psych-071112-054629 22994921

[B23] Haß L. H. Müller M. A. Vergauwe S. (2015). Tournament incentives and corporate fraud. *J. Corp. Finance* 34 251–267. 10.1016/j.jcorpfin.2015.07.008

[B24] Holmstrom B. (1989). Agency costs and innovation. *J. Econ. Behav. Organ.* 12 305–327. 10.1016/0167-2681(89)90025-5

[B25] Huang J. Jain B. A. Kini O. (2019). Industry Tournament Incentives and the Product-Market Benefits of Corporate Liquidity. *J. Financ. Quant. Anal.* 54 829–876. 10.1017/S0022109018000704

[B26] Kale J. R. Reis E. Venkateswaran A. (2009). Rank-Order Tournaments and Incentive Alignment: the Effect on Firm Performance. *J. Finance* 64 1479–1512. 10.1111/j.1540-6261.2009.01470.x

[B27] Khan T. M. Bai G. Fareed Z. Quresh S. Khalid Z. Khan W. A. (2021). CEO Tenure, CEO Compensation, Corporate Social and Environmental Performance in China: the Moderating Role of Coastal and Non-coastal Areas. *Front. Psychol.* 11:574062. 10.3389/fpsyg.2020.574062 33551900 PMC7862113

[B28] Kim T.-Y. Leung K. (2007). Forming and reacting to overall fairness: a cross-cultural comparison. *Organ. Behav. Hum. Decis. Process.* 104 83–95. 10.1016/j.obhdp.2007.01.004

[B29] Kitayama S. Uskul A. K. (2011). Culture, Mind, and the Brain: current Evidence and Future Directions. *Annu. Rev. Psychol.* 62 419–449. 10.1146/annurev-psych-120709-145357 21126182

[B30] Lazear E. P. Rosen S. (1981). Rank-Order Tournaments as Optimum Labor Contracts. *J. Polit. Econ.* 89 841–864. 10.1086/261010

[B31] Lewis R. S. Goto S. G. Kong L. L. (2008). Culture and Context: east Asian American and European American Differences in P3 Event-Related Potentials and Self-Construal. *Pers. Soc. Psychol. Bull.* 34 623–634. 10.1177/0146167207313731 18413894

[B32] Ma M. Pan J. Stubben S. R. (2019). The Effect of Local Tournament Incentives on Firms’ Performance, Risk-Taking Decisions, and Financial Reporting Decisions. *Account. Rev.* 95 283–309. 10.2308/accr-52506

[B33] Mas A. (2006). Pay, Reference Points, and Police Performance. *Q. J. Econ.* 121 783–821. 10.1162/qjec.121.3.783

[B34] Park S. H. Westphal J. D. (2013). Social Discrimination in the Corporate Elite: how Status Affects the Propensity for Minority CEOs to Receive Blame for Low Firm Performance. *Adm. Sci. Q.* 58 542–586. 10.1177/0001839213509364

[B35] Rasool S. F. Chin T. Wang M. Asghar A. Khan A. Zhou L. (2021). Exploring the role of organizational support, and critical success factors on renewable energy projects of Pakistan. *Energy* 242 122765. 10.1016/j.energy.2021.122765

[B36] Rasool S. F. Samma M. Wang M. Zhao Y. Zhang Y. (2019). How Human Resource Management Practices Translate Into Sustainable Organizational Performance: the Mediating Role of Product, Process and Knowledge Innovation. *Psychol. Res. Behav. Manage.* 12 1009–1025. 10.2147/PRBM.S204662 31802958 PMC6830386

[B37] Ren Z. (2012). Spirituality and Community in Times of Crisis: encountering Spirituality in Indigenous Trauma Therapy. *Pastoral Psychol.* 61 975–991. 10.1007/s11089-012-0440-5

[B38] Romer P. M. (1986). Increasing Returns and Long-Run Growth. *J. Polit. Econ.* 94 1002–1037.

[B39] Rong P. Wang C. (2021). CEO Turnover, Leadership Identity, and TMT Creativity in a Cross-Cultural Context. *Front. Psychol.* 12:610526. 10.3389/fpsyg.2021.610526 34512428 PMC8424047

[B40] Ruff C. C. Fehr E. (2014). The neurobiology of rewards and values in social decision making. *Nat. Rev. Neurosci.* 15 549–562. 10.1038/nrn3776 24986556

[B41] Scarpa M. P. Di Martino S. Prilleltensky I. (2021). Mattering Mediates Between Fairness and Well-being. *Front. Psychol.* 12:744201. 10.3389/fpsyg.2021.744201 34858276 PMC8630584

[B42] Scholl R. W. Cooper E. A. McKenna J. F. (1987). Referent Selection in Determining Equity Perceptions: differential Effects on Behavioral and Attitudinal Outcomes. *Pers. Psychol.* 40 113–124. 10.1111/j.1744-6570.1987.tb02380.x

[B43] Song J. Price D. J. Guvenen F. Bloom N. von Wachter T. (2019). Firming Up Inequality. *Q. J. Econ.* 134 1–50. 10.1093/qje/qjy025

[B44] Stallen M. Sanfey A. G. (2013). The Cooperative Brain. *Neuroscientist* 19 292–303. 10.1177/1073858412469728 23300215

[B45] Tekleab A. G. Bartol K. M. Liu W. (2005). Is It Pay Levels or Pay Raises That Matter to Fairness and Turnover? *J. Organ. Behav.* 26 899–921. 10.1002/job.352

[B46] Torre E. D. Pelagatti M. Solari L. (2015). Internal and external equity in compensation systems, organizational absenteeism and the role of explained inequalities. *Hum. Relat.* 68 409–440. 10.1177/0018726714528730

[B47] Tricomi E. Rangel A. Camerer C. F. O’Doherty J. P. (2010). Neural evidence for inequality-averse social preferences. *Nature* 463 1089–1091. 10.1038/nature08785 20182511

[B48] Tu W. (2005). Cultural China: the Periphery as the Center. *Daedalus* 134 145–167.

[B49] Varnum M. E. W. Hampton R. S. (2017). Cultures differ in the ability to enhance affective neural responses. *Soc. Neurosci.* 12 594–603. 10.1080/17470919.2016.1209239 27420406

[B50] Wang J. Deng J. (2021). Research on the Effect of Executive Incentive Institutional Innovation on the Cost of Equity—Evidence From Chinese Listed Companies. *Front. Psychol.* 12:686955. 10.3389/fpsyg.2021.686955 34168600 PMC8217634

[B100] Xu Y. (2012). *Thus Spoke the Master(Chinese-English).* Beijing: Zhonghua Book Company.

[B51] Yang S. Wu H. (2021). The Performance Impact of New Ventures in Working Environment and Innovation Behavior From the Perspective of Personality Psychology. *Front. Psychol.* 12:734014. 10.3389/fpsyg.2021.734014 34803812 PMC8595093

[B52] Yu Z. Xiao Y. Li J. (2021). Firm-level perception of uncertainty and innovation activity: textual evidence from China’s A-share market. *Pac. Basin Financ. J.* 68:101555. 10.1016/j.pacfin.2021.101555

